# Alternative splicing event associated with immunological features in bladder cancer

**DOI:** 10.3389/fonc.2022.966088

**Published:** 2023-01-05

**Authors:** Xinbo Yu, Bixian Luo, Jianwei Lin, Yu Zhu

**Affiliations:** ^1^ Department of Urology, Ruijin Hospital, Shanghai Jiao Tong University School of Medicine, Shanghai, China; ^2^ Department of General Surgery, Ruijin Hospital, Shanghai Jiao Tong University School of Medicine, Shanghai, China

**Keywords:** alternative splicing, bladder cancer, risk score, nomogram, immune checkpoint

## Abstract

Bladder cancer (BLCA) is the most prevalent urinary tumor with few treatments. Alternative splicing (AS) is closely related to tumor development and tumor immune microenvironment. However, the comprehensive analysis of AS and prognosis and immunological features in BLCA is still lacking. In this study, we downloaded RNA-Seq data and clinical information from The Cancer Genome Atlas (TCGA) database, and AS events were acquired from the TCGA Splice-seq. A total of eight prognostic AS events (C19orf57|47943|ES, ANK3|11845|AP, AK9|77203|AT, GRIK2|77096|AT, DYM|45472|ES, PTGER3|3415|AT, ACTG1|44120|RI, and TRMU|62711|AA) were identified by univariate analysis and least absolute shrinkage and selection operator (LASSO) regression analysis to construct a risk score model. The Kaplan–Meier analysis revealed that the high-risk group had a worse prognosis compared with the low-risk group. The area under the receiver operating characteristic (ROC) curves (AUCs) for this risk score model in 1, 3, and 5 years were 0.698, 0.742, and 0.772, respectively. One of the prognostic AS event-related genes, TRMU, was differentially expressed between tumor and normal tissues in BLCA. The single-sample gene set enrichment analysis (ssGSEA) and CIBERSORT algorithm showed that both the risk score model and TRMU were significantly associated with tumor immune microenvironment and immune status (immune cells, immune-related pathway, and immune checkpoint) in BLCA patients. The TIMER database confirmed the relationship between the expression of TRMU and immune cells and checkpoint genes. Furthermore, Cytoscape software 3.8.0 was used to construct the regulatory network between AS and splicing factors (SFs). Our study demonstrated that AS events were powerful biomarkers to predict the prognosis and immune status in BLCA, which may be potential therapeutic targets in BLCA.

## Introduction

Bladder cancer (BLCA) is a common malignancy in the human urinary system, with almost 64,280 new cases and 12,260 deaths in the United States in 2021 (1). The most common subtype of BC is urothelial carcinomas, accounting for 90% of all cases. Among urothelial carcinoma, approximately 75% of cases are diagnosed with non-muscle invasive bladder cancer (NMIBC), and the remaining cases are muscle-invasive bladder cancer (MIBC) (2). Patients with NMIBC have a high incidence of recurrence and may progress to MIBC and metastatic disease. The prognosis of patients with MIBC is poor due to the high risk of metastasis and weak therapeutic reactivity (3).

Immunotherapy has emerged as an effective treatment for various cancer types. Intravesical bacillus Calmette–Guérin (BCG) was recognized as a well-known immunotherapy since the 1970s, which remains the gold standard treatment for patients with NMIBC (4). Over the last few years, immune checkpoint inhibitors (ICIs) such as cytotoxic T-lymphocyte-associated antigen 4 (CTLA-4) inhibitors, programmed death 1 (PD-1) receptor inhibitors, and programmed death ligand-1 (PD-L1) inhibitors have been approved by the Food and Drug Administration (FDA) in MIBC patients (5). The use of ICI has dramatically altered the treatment for BLCA, which has shown good and long-lasting results since 2016. However, only 20%–30% of BLCA patients show full immunotherapeutic responses to ICI therapy, and reliable predictive biomarkers are lacking (6). Therefore, accurate and effective biomarkers are critical to predict the ICI treatment efficacy and prognosis in BLCA patients. Previous studies have demonstrated that tumor immune microenvironment (TIME) phenotype biomarkers may be effective for predicting the efficacy of ICIs (7, 8). The TIME includes many types of cells (immune cells, vessel cells, fibroblasts, and the extracellular matrix), which have complex biological relationships with tumor cells and could drive tumor repression or progression across tumor patients (9). Despite promising results, the efficacy rate of these biomarkers still lacks enough stability and requires validation. Therefore, it is worthwhile to explore additional biomarkers to improve the predictive accuracy of ICI treatment for BLCA patients.

Alternative splicing (AS) is a highly complex posttranscriptional process that enhances protein diversity and the progression of cancer. There are seven basic AS events: alternate acceptor sites (AA), alternate donor sites (AD), alternate terminator (AT), alternate promoter (AP), mutually exclusive exons (ME), exon skip (ES), and retained intron (RI) (10). Approximately 95% of human genes are modified by AS, which increases the complexity of mRNA isoforms and thus enriches the protein diversity. Dysregulation of AS events participate in the development of cancer and its progression (11). Moreover, growing evidence demonstrated the correlation between AS events and tumor immune microenvironment (12). Hence, whether AS events can serve as potential therapeutic targets and biomarkers for predicting immunotherapeutic responses in BLCA patients remains unknown.

In this research, we constructed a prognostic prediction model derived from prognostic AS events based on The Cancer Genome Atlas (TCGA) database and TCGA SpliceSeq database. Subsequently, the BLCA patients were divided into the high- and low-risk groups according to the median cutoff risk score, the nomogram was generated, and immune microenvironment analysis was performed. Furthermore, we identified differentially expressed genes (DEGs) related to prognostic AS events between BLCA patients and normal bladder samples and then performed immune microenvironment analysis again. Finally, the AS splicing factor (SF) network was generated to show potential regulatory mechanisms. These analyses suggest that AS events may serve as biomarkers to predict the immunotherapeutic responses and prognosis in BLCA patients

## Materials and methods

### Data acquisition and processing

The RNA sequencing data and clinical information of BLCA patients and normal bladder samples were obtained from TCGA data portal (https://tcga-data.nci.nih.gov/tcga/). The clinical information of BLCA patients from TCGA is shown in **Table 1**. AS event data were also downloaded from TCGA SpliceSeq database (https://bioinformatics.mdanderson.org/TCGASpliceSeq/); the filters of splice events were set as 75% of samples with percent spliced in (PSI) value.

**Table 1:** Clinical information of BLCA patients from TCGA.

		TCGA n = 412
Age	≤65 >65 Unknown	162 250 0
Gender	Female Male	108 304
Stage	Stage 0 Stage I–II Stage III–IV Unknown	0 133 277 2
T	T0 T1–2 T3–4 Tx Unknown	1 123 255 1 32
M	M0 M1 Mx Unknown	196 11 202 3
N	N0 N1–2 N3 Nx Unknown	239 123 8 36 6

BLCA, bladder cancer; TCGA, The Cancer Genome Atlas.

### Development of the risk model related to AS events

The BiocManager R packages were utilized to merge the clinical information and AS events, and a univariate Cox regression analysis was applied to reveal the relationship between overall survival (OS) and AS events, where p < 0.05 was considered statistically significant. The UnicoxUpset plot was constructed to display the intersection between gene and prognostic AS events. The least absolute shrinkage and selection operator (LASSO) regression analysis was further used to improve the risk model, and the risk score was calculated based on the PSI value. Eight prognostic AS events were screened to build the risk score model. The patients were separated into a high-risk subgroup and a low-risk subgroup according to the median cutoff risk score.

### Assessment of prognostic model and construction of nomogram

With the use of the survminer and pheatmap R packages, the survival analysis and pheatmap were used to evaluate the differences in patients’ survival and AS event-associated genes between the high- and low-risk groups. The independence of the risk score along with clinical features was also evaluated by using univariate and multivariate Cox regression forest plots. The 1-, 3-, and 5-year receiver operating characteristic (ROC) curve was used to evaluate the survival prediction ability of the prognostic model compared with clinical features. Furthermore, the nomogram was constructed for inspecting the probability of 1-, 3-, and 5-year OS of the BLCA patients, and the calibration curve was generated to visualize the discrimination of the nomogram.

### Comparison of the tumor immune microenvironment between subgroups

The tumor immune microenvironment scores (Stromal Score, Immune Score, ESTIMATE Score, and tumor purity) in TCGA-BLCA patients were calculated using the “ESTIMATE” R package (13). Next, the violin plots were drawn to analyze the difference in tumor immune microenvironment scores between the high- and low-risk groups.

### Immune activity analysis

The differences in immune cell infiltration in the high- and low-risk groups were analyzed using the R package “CIBERSORT”, where p < 0.05 was regarded as a significant result (14). The single-sample gene set enrichment analysis (ssGSEA) was employed to explore the difference in infiltrating score of immune cells and immune-related pathways between two subgroups (15). A heatmap was also plotted to visually assess the differential expression of immune cells, immune-related pathways, and tumor immune microenvironment between two subgroups. In addition, the differences in immune checkpoint genes in the high- and low-risk groups were analyzed.

### Analysis of differentially expressed prognostic AS events

The “Limma” package in R was utilized to screen differentially expressed prognostic AS events related to genes in tumors versus normal samples (16). The condition of this differential analysis was set as follows: |log2FC| > 0.8 and p < 0.05 (FC, fold change). The level of TRMU was verified by the Gepia2 database (http://gepia2.cancer-pku.cn/#index). The analysis of immune cells, immune-related pathways, tumor immune microenvironment, and immune checkpoints used the above-mentioned method. The gene expression and immune infiltration were visually assessed by TIMER (http://timer.cistrome.org), which is a public database for the analysis of immune infiltration in various types of cancer.

### Construction of SF-AS regulatory network

In this study, a total of 404 SF genes were obtained from other literature (17). We used Spearman’s correlation method to evaluate the correlation between SF and survival-related AS events, where the correlation coefficient > 0.65 and p < 0.001 were considered statistically significant relationships. The SF-AS regulatory network was constructed and drawn by the software of Cytoscape (version 3.8.0) (18).

### Statistical analysis

The Wilcoxon test was performed to compare two groups, whereas the Kruskal–Wallis test was used to compare more than two groups. Risk scores, clinical variables, immune cell infiltrating extent, and immune checkpoints were performed *via* Pearson’s correlation test. p < 0.05 was considered statistically significant. All statistical analyses were done using R 4.10 (https://www.r-project.org).

## Results

### Identification of prognostic AS events in BLCA

**Figure 1:**
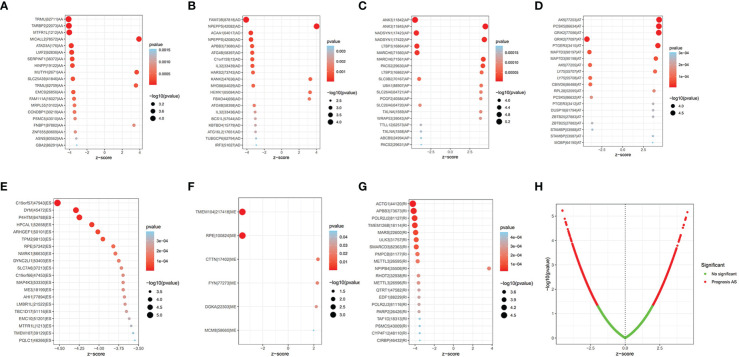
The prognostic AS events. **(A–G)** The bubble plot of the 20 most relevant prognostic AS events in different types of AS events, except ME. **(H)** The volcano plots of prognostic AS events. AS, alternative splicing; ME, mutually exclusive exons.

The Upset plot displayed a total of 17,739 AS events from 9,415 parent genes, the most frequent splicing type was ES, and the least frequent type was ME (**Supplementary Figure S1A**). Univariate Cox regression analysis of these AS events revealed that 1,972 AS events from 1,755 parental genes were significantly related to the OS (p < 0.05), as shown in **Figure S1B**; ES still had the highest percentage (28.45%) among AS events related to OS. The top 20 AS events associated with OS of each type are clearly exhibited in **Figure 1**. Volcano plots show the distribution of AS events with and without significant relation with OS (**Figure 1**).

### Construction and assessment of risk model

**Figure 2:**
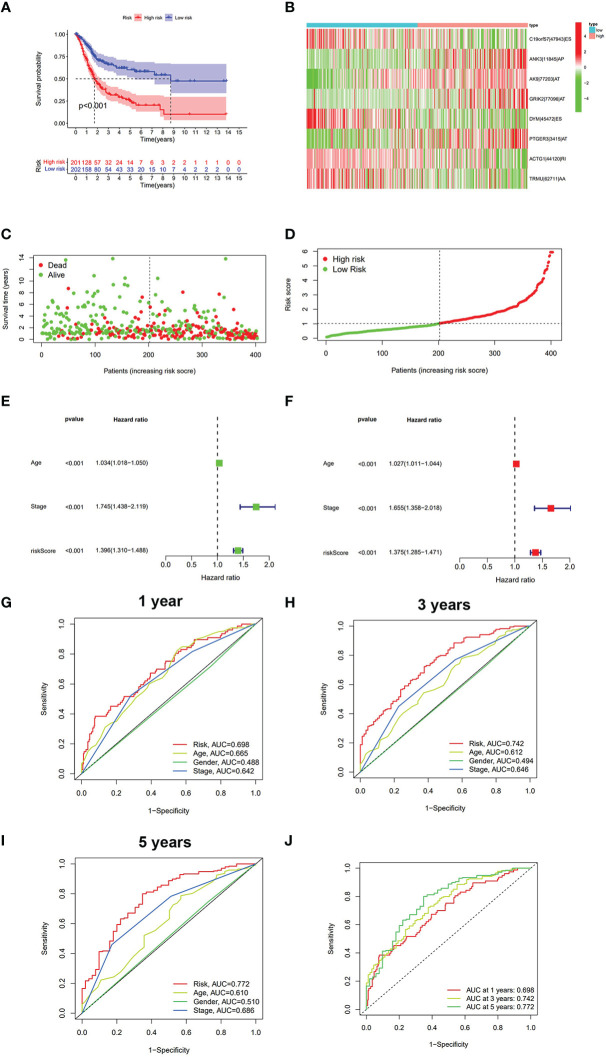
The analysis of risk score model in BLCA. **(A)** Kaplan–Meier survival analysis of OS between the low-risk group and the high-risk group. **(B)** Heatmap of eight prognostic AS events between the low-risk group and the high-risk group. **(C)** Survival state diagram of BLCA; green dots represent survival, and red dots represent death. **(D)** The distribution of risk score among BLCA patients. **(E)** Univariate analysis of risk score and other clinical parameters. **(F)** Multivariate analysis of risk score and other clinical parameters. **(G)** ROC curve of risk score and other clinical parameters at 1 year. **(H)** ROC curve of risk score and other clinical parameters at 3 years. **(I)** ROC curve of risk score and other clinical parameters at 5 years. **(J)** ROC curve of risk score at 1, 3, and 5 years. BLCA, bladder cancer; OS, overall survival; ROC, receiver operating characteristic.

The LASSO regression was first performed to find the most significant prognostic AS events and prevent overfitting of the model. The results of the LASSO regression analysis are displayed in **Figure S2**. Multivariate Cox regression was then performed to develop the risk score model (C19orf57|47943|ES, ANK3|11845|AP, AK9|77203|AT, GRIK2|77096|AT, DYM|45472|ES, PTGER3|3415|AT, ACTG1|44120|RI, and TRMU|62711|AA) and calculate the risk score. BLCA patients were divided into the high-risk group and low-risk group according to the median cutoff risk score. The Kaplan–Meier survival analysis showed the low-risk group was associated with better survival outcomes as compared with the high-risk group (p < 0.001) (**Figure 2A**). The distribution of risk score, survival status, and expression heatmap of PSI value is shown in **Figures 2B–D**.

**Figure 3:**
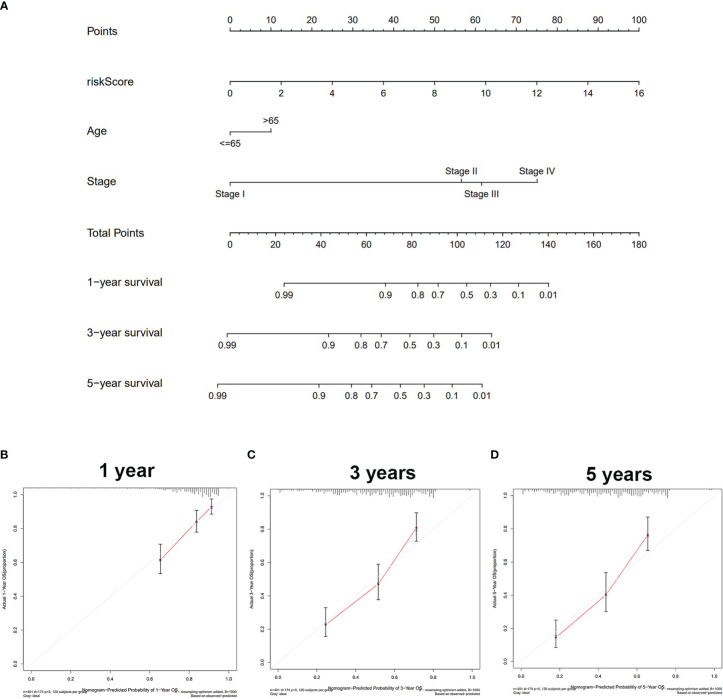
Construction and validation of the nomogram. **(A)** The nomogram based on riskScore, age, and stage to predict the 1-, 3-, and 5-year survival in BLCA patients. **(B)** One-year nomogram calibration. **(C)** Three-year nomogram calibration. **(D)** Five-year nomogram calibration. BLCA, bladder cancer.

The univariate/multivariate Cox regression analyses revealed that risk score was an independent prognostic indicator for BLCA patients, as well as other clinical parameters (age and stage) (**Figures 2E, F**). Subsequently, the ROC curve was used to assess the prediction accuracy of risk score and other clinical parameters in predicting the 1-, 3-, and 5-year survival probability of BLCA patients. As shown in **Figures 2G–J**, the AUC value of the risk score was larger than that of other clinical parameters and reached 0.698, 0.742, and 0.772, respectively. Furthermore, we analyzed the relationship between risk score and other clinical parameters (age, gender, T, M, N, and clinical stage), and the results showed that there were several significant differences between risk score and subgroups of clinical parameters (**Supplementary Figure S3**). The risk score increased significantly with tumor grade (most p < 0.05, **Figure S3C**), T category (most p < 0.05, **Figure S3D**), and M category (most p < 0.05, **Figure S3E**), which meant that the risk score can reflect the progression of BLCA. Based on three independent prognostic indicators, the nomogram was constructed to predict the 1-, 3-, and 5-year survival probability of BLCA patients (**Figure 3A**). In addition, calibration curves indicated the good agreement of this nomogram for the probability of survival at 1, 3, or 5 years (**Figures 3B–D**). These results show that the nomogram in our study can predict the actual survival outcome well.

### Prognostic AS events were associated with tumor immune microenvironment and immune activity

**Figure 4:**
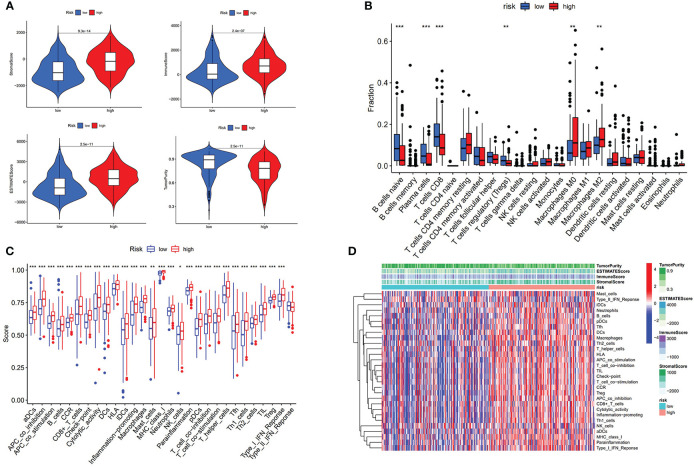
Risk score associated with immunological features. **(A)** Comparison of stromal score, immune score, estimate score, and tumor purity between low-risk group and high-risk group. **(B)** Comparison of immune cells is displayed in boxplot. **(C)** Score of immune-related pathway. **(D)** Heatmap of immune features of two subgroups. * p < 0.05, **p < 0.01, ***p < 0.001.

To comprehensively analyze the immune features based on prognostic AS events, the tumor immune microenvironment scores between the high- and low-risk groups were compared. The high-risk group was found to have a higher level of stromalScore, immuneScore, and ESTIMATEScore and a lower level of tumor purity (p < 0.001) (**Figure 4A**). CIBERSORT was then performed to analyze the relationship between risk score and immune cells, and it was found that patients in the high-risk group were positively associated with infiltration of innate immune cells (macrophages M0 and macrophages M2) (p < 0.01) and negatively associated with infiltration of adaptive immune cells (B cells naïve, T cells CD8, and so on) (p < 0.01) (**Figure 4B** and **Supplementary Figure S4**). ssGSEA was used to detect the immune-related pathway and cells between the low-risk and the high-risk group. As shown in **Figure 4C**, there was a significant difference in most of the immune-related pathways and cells between the two subgroups. The heatmap of the above results is shown in **Figure 4D**.

**Figure 5:**
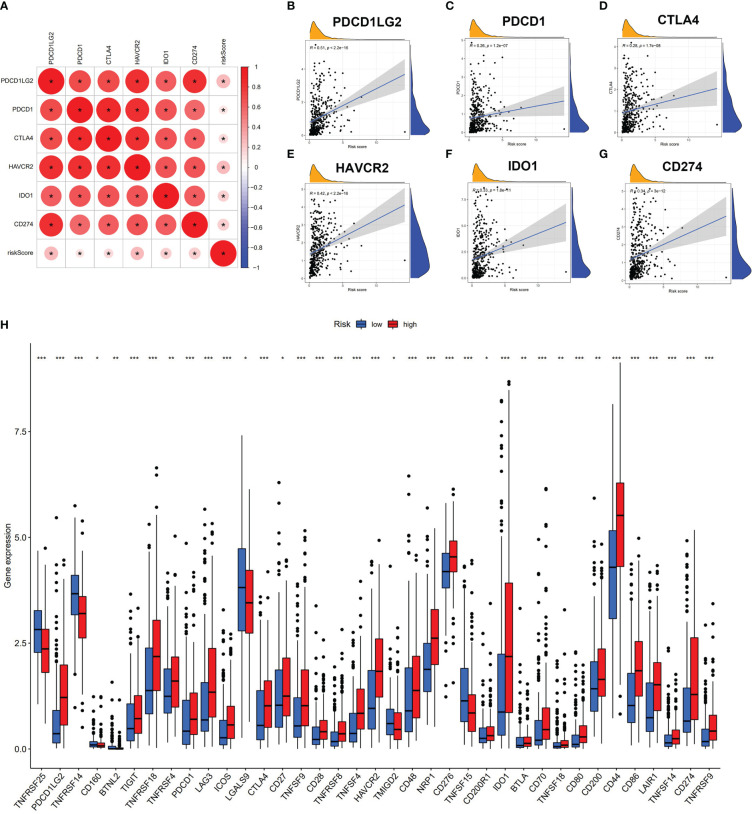
Risk score associated with immune checkpoint genes. **(A–G)** Correlation analysis of risk score and six hub immune checkpoint genes (CD274, PDCD1, PDCD1LG2, CTLA4, HAVCR2, and IDO1). **(H)** Comparison of immune checkpoint genes of two subgroups. * p < 0.05, **p < 0.01, ***p < 0.001.

We further revealed that the risk score was positively correlated with six hot immune checkpoint genes (CD274, PDCD1, PDCD1LG2, CTLA4, HAVCR2, and IDO1); the results are shown in **Figures 5A–G**. Finally, we compared the relative quantity of immune checkpoint genes between the two subgroups, and we found that the relative quantity of most (31/47) immune checkpoint genes was significantly higher in the high-risk group (**Figure 5H**), suggesting that prognostic AS events might act as a non-negligible and unfavorable factor in immunotherapy treatment. In total, these results suggested that our risk score model may reflect tumor immune microenvironment, infiltration of immune cells, immune-related signaling pathway, and immune checkpoint, which may provide a potential target for immunotherapy in BLCA patients.

### Correlation of TRMU with immune activity

**Figure 6:**
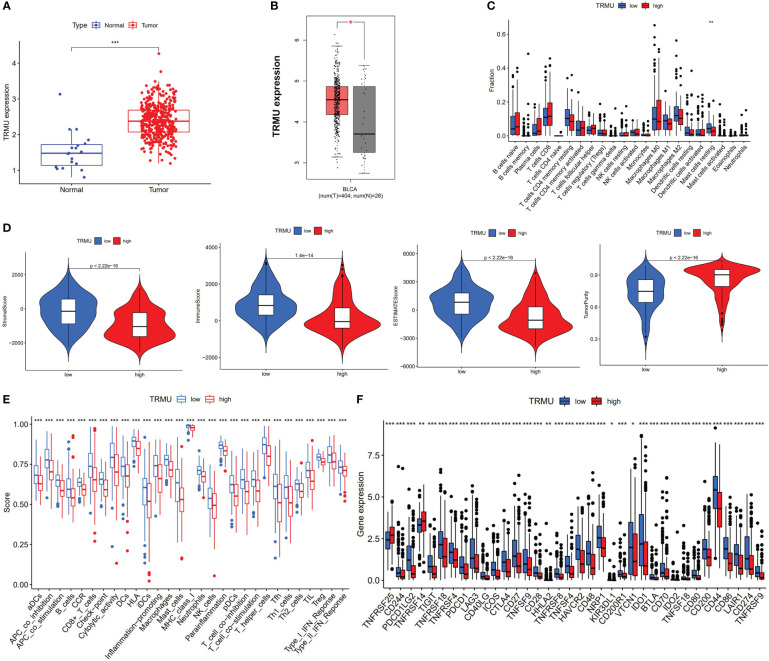
Correlation between TRMU and immunological features. **(A)** The expression of TRMU between normal tissues and tumor tissues. **(B)** The expression of TRMU verified by GEPIA2 database. **(C)** Comparison of immune cells between low-TRMU group and high-TRMU group. **(D)** Comparison of stromal score, immune score, estimate score, and tumor purity between low-TRMU group and high-TRMU group. **(E, F)** Comparison of immune-related pathway and immune checkpoint genes among two subgroups. * p < 0.05, **p < 0.01, ***p < 0.001.

We performed the differential expression analysis (|log2FC| > 0.8 and p < 0.05) from risk score based on eight prognostic AS events between normal tissues and tumor tissues. TRMU (logFC = 0.84, p < 0.001) was found to be overexpressed in BLCA tissues (n = 414) compared to normal tissues (n = 19) (**Figure 6A**). The GTEx database contains the RNA sequencing of the bladder (n = 9), and the expression of TRMU was verified in the GEPIA2 database, which matches TCGA and GTEx data (404 tumors *vs.* 29 normal tissues, **Figure 6B**). In the TCGA-BLCA cohort, patients were divided into a high-TRMU group and a low-TRMU group according to the optimal cutoff expression value of TRMU. Our results indicated that the tumor immune microenvironment scores were markedly different between the two subgroups (**Figure 6D**). In addition, the result of ssGSEA demonstrated that the low-TRMU group was related to a variety of immune regulatory signaling pathways (**Figure 6E**).

**Figure 7:**
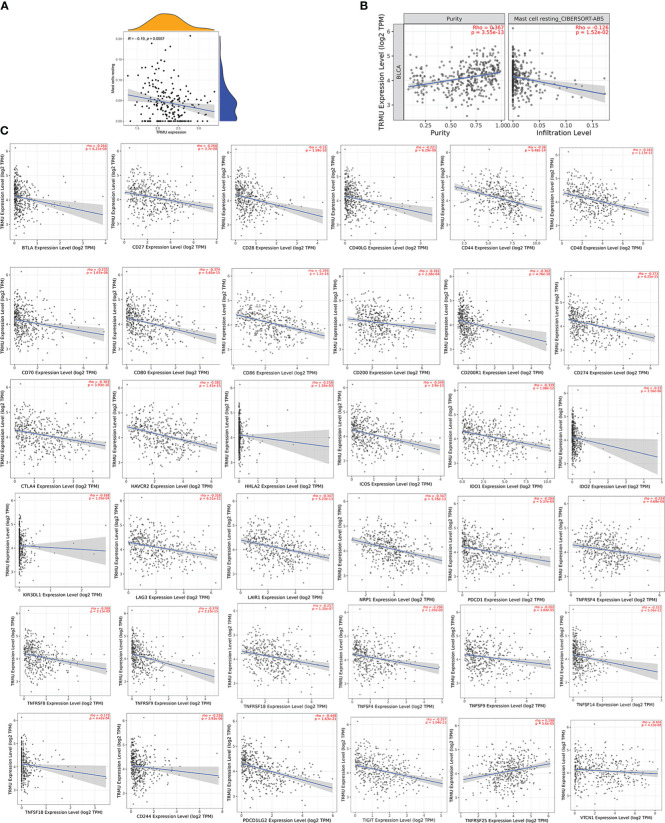
Correlation between TRMU and immune cells and immune checkpoint gene. **(A)** Relationship between TRMU and mast cell resting. **(B)** Correlation of TRMU and mast cell resting in TIMER database. **(C)** Correlation of TRMU and immune checkpoint gene in TIMER database.

Based on the CIBERSORT algorithm, TRMU was negatively associated with infiltration of mast cell resting (r = −0.19, p < 0.01) (**Figures 6C**, **7A**). TRMU was negatively related to 35/47 immune checkpoint genes whereas only positively related to TNFRSF25 (p < 0.05) (**Figure 6F**). These results were verified again from the TIMER database (**Figures 7B, C**).

### Construction of SF-AS regulatory network

**Figure 8:**
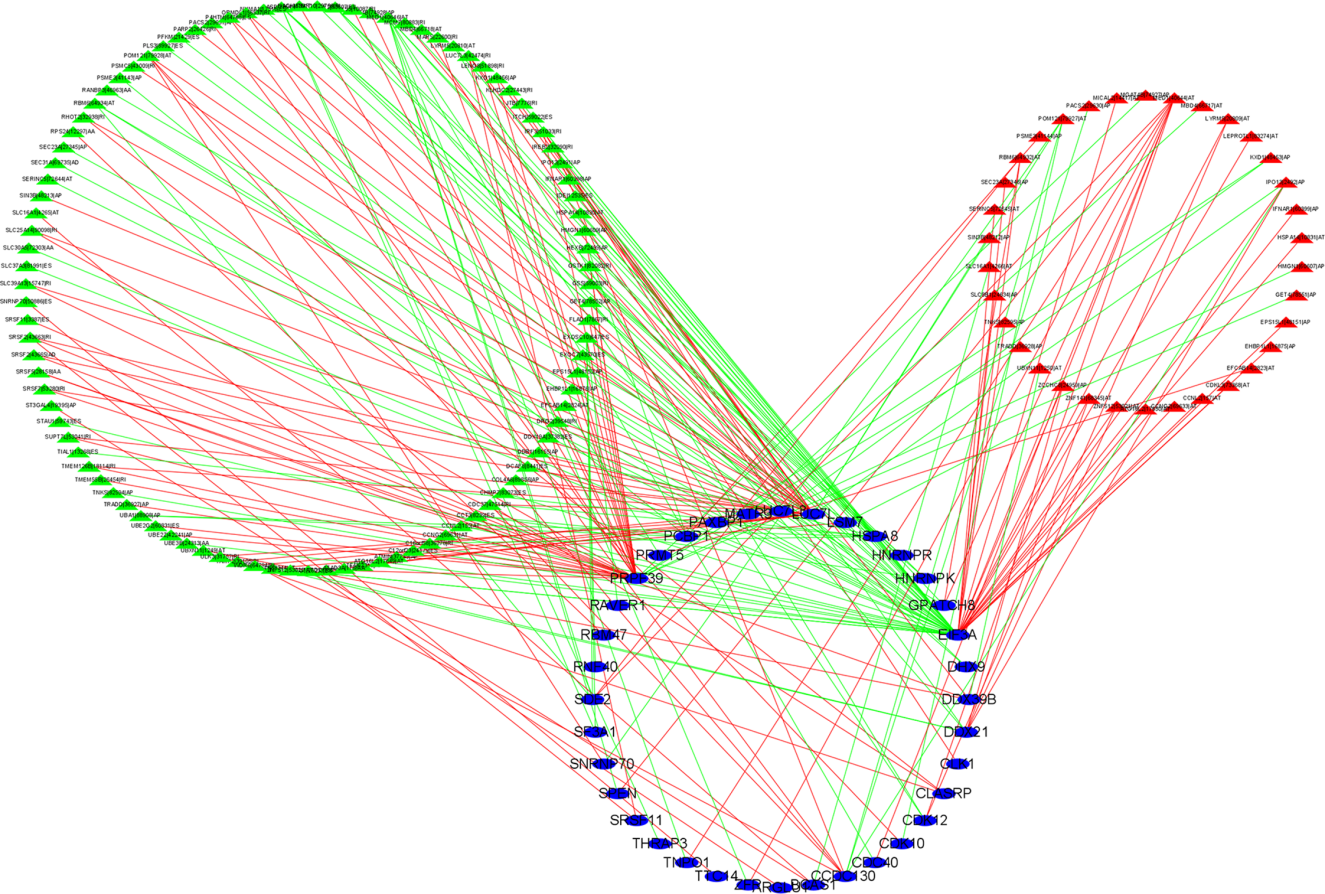
Construction of alternative splicing events and splicing factors regulatory network. Blue ellipse represents splicing factors, green/red triangle represents favorable/adverse alternative splicing events, and green/red line represents positive/negative regulation. Cor > 0.65, p < 0.001.

To explore the potential regulatory relationship between OS-related AS events and SF genes in BLCA patients, Pearson’s correlation analysis was performed. The significant association was regarded as correlation coefficients greater than 0.65 and p-value less than 0.001. We identified a total of 36 SFs (blue ellipse) that were significantly correlated with 34 adverse AS events (red triangle) and 99 favorable AS events (green triangle). The correlation network was shown in **Figure 8**. Interestingly, the adverse AS events only comprised two types of AS events: AP and AT. In addition, SFs were positively (red line) related with 122 AS events and negatively (green line) with 93 AS events. In our regulatory network, the top 4 most significant nodes were termed hub SFs or AS events (**Supplementary Table S1**), including three upregulated AS events (MED1|40644|AT, SRSF2|43663|RI, and RBM6|64932|AT) and three SFs (EIF3A, PRPF39, and LUC7L3).

## Discussion

BLCA is one of the most common urinary tumors, most of which originate from the urothelium. An increasing number of studies have demonstrated that immunotherapy has a considerable role in the treatment of BLCA, especially for the use of immune checkpoint (PD-1, PD-L1, and CTLA-4) inhibitors (19–22). It is estimated that only a subset of BLCA patients have durable immunotherapeutic responses to ICIs (23). Moreover, the substantial costs of ICIs become a heavy burden for patients. There is an urgent need for novel biomarkers to identify the immunotherapeutic responses among BLCA patients. AS was considered an essential mechanism of mRNA variation and proteomic diversity of many cancers (24). Increasing studies have provided strong evidence to support that AS could be an effective biomarker for predicting prognosis and immunotherapy immunotherapeutic outcomes in a series of tumors, such as hepatocellular carcinoma (25), pancreatic adenocarcinoma (26), and breast cancer (27). However, the relationship between AS events and immunological features in BLCA is still under investigation.

In this study, AS events data were obtained from the SpliceSeq database and clinical data from TCGA database to analyze the relationship between AS events and BLCA. After univariate Cox regression analysis, a total of 1,972 AS events related to OS were identified, with the highest number of ES type, and 1,755 parental genes were involved. Dual analyses of LASSO regression and multivariate Cox regression were performed to estimate the risk score model (C19orf57|47943|ES, ANK3|11845|AP, AK9|77203|AT, GRIK2|77096|AT, DYM|45472|ES, PTGER3|3415|AT, ACTG1|44120|RI, and TRMU|62711|AA). It was found that patients with higher risk scores were associated with poor prognoses, and risk scores could act as independent indicators in BLCA. Furthermore, the AUC values of the risk score model were more reliable than other clinical parameters, with a high efficacy of 0.772 for 5-year OS, indicating effective prediction accuracy for the prognosis of BLCA. The validation results of the nomogram based on risk score and other independent prognostic factors (age and stage) verified good accuracy in predicting the survival outcome.

In the past few years, the relevance of AS and inflammatory microenvironment has fostered new therapeutic strategies in human cancer (28). We conducted TIMER database, ESTIMATE algorithm, ssGSEA method, and CIBERSORT analyses to reveal the relationship between AS and TIME in BLCA. The results presented that patients with higher risk scores were characterized by low tumor purity associated with high immune scores and stromal scores. Previous studies established a connection between low tumor purity and poor prognosis (29–31), which was consistent with our experimental results. The results of the CIBERSORT algorithm showed that high-risk patients were associated with high infiltration of macrophages (M0, M2). Studies have shown that a high number of M2-like macrophages provide support for tumor progression and cancer metastasis in patients with BLCA (32–34). Moreover, patients with lower risk scores were positively associated with infiltration of adaptive immune cells including B cells naïve, plasma cells, CD8 T cells, and regulatory T cells. In addition, our results of the ssGSEA method revealed that high-risk-score patients were closely related to immune-related pathways. Furthermore, a high risk score was positively associated with the expression levels of six key immune checkpoint genes (CD274, PDCD1, PDCD1LG2, CTLA4, HAVCR2, and IDO1) and other related immune checkpoint genes, which indicates that risk score might be a target to distinguish the immunotherapy efficacy among BLCA patients. These results suggest that a high risk score might have a stronger immune response, which contributes to the repression of tumors. We also observed that the expression levels of PD-1 (PDCD1), PD-L1 (CD274), and CTLA4 in the high-risk-score group were higher than those of the low-risk-score group. Similarly, BLCA patients with higher risk scores are more sensitive to ICIs, including inhibitors of PD-1 (PDCD1), PD-L1 (CD274), and CTLA4. In general, we constructed a risk model that serves as an effective biomarker for strategizing tailored immunotherapy in BLCA patients.

TRMU is a nuclear gene that encodes a highly conserved mitochondrial protein that participates in mitochondrial tRNA modifications (35). Studies have reported that mutations in TRMU cause acute infantile liver failure (36, 37). However, little research revealed the role of TRMU in tumors, especially in BLCA. Our results showed that TRMU was significantly upregulated in BLCA tumor tissues compared with normal tissues. The expression of TRMU was negatively associated with the immune microenvironment, the abundance of most immune-related pathways, infiltration of mast cell resting, and almost all immune checkpoint genes except TNFRSF25. This study also used the TIMER database to assess the relationship between TRMU and immunological characteristics, which improve the efficacy of our research. The SF-AS regulatory network was constructed to reveal the potential regulatory mechanism. The relationship between AS events and SFs was regarded as multiple interactions instead of “one-to-one correspondence”, providing a target of immunotherapy in BLCA.

SF-AF regulatory network reveals the potential regulatory relationship between SFs and AS. We identified three key SFs. EIF3A (eukaryotic translation initiation factor 3a) is an essential functional entity in ribosome establishment and translation initiation. EIF3a is overexpressed in urinary bladder cancer, and high EIF3a expression was linked to longer overall survival rates of patients with low-grade tumors (38). PRPF39 and LUC7L3 were never been identified as potential SFs in the development of BLCA.

Compared with existing studies (39, 40) that explored the AS event signature in BLCA, this study has taken into consideration the relationship of AS prognostic signature with TIME. In addition, the established risk score model with high accuracy further accurately predicted immune status and ICI treatment in patients with BLCA. Finally, this work first placed emphasis on the immunological roles of differentially expressed prognostic AS event-related gene TRMU in BLCA, highlighting the association of the gene expression of prognostic AS event to the outcome of ICI treatment.

However, our research has some limitations. First, this study only used the data from the public TCGA database without verification in another database. Second, the mechanisms of AS events and SFs in BLCA need to be experimentally verified. Third, our study is based on pure bioinformatics analysis and lacks experimental validation.

## Conclusion

In conclusion, our study first performed systematic analyses between AS events, prognosis, and immune features in BLCA patients. The risk score model based on eight prognostics AS events was constructed to predict the clinical outcome of BLCA patients. Both the risk score model and TRMU were closely linked to the tumor immune microenvironment, immune cells, immune-related pathways, and immune checkpoint genes. These findings provide insight that AS events could serve as therapeutic targets in BLCA patients.

## Data availability statement

The datasets presented in this study can be found in online repositories. The names of the repository/repositories and accession number(s) can be found in the article/**Supplementary Material**.

## Author contributions

This article was written by XY. BL and LJ helped to modify the relevant R language codes and related pictures. YZ provided guidance to the manuscript preparation and research ideas for the writing of the manuscript. All authors contributed to the article and approved the submitted version.
