# Supplementary material for: Alternative splicing event associated with immunological features in bladder cancer
Source: Front Oncol. 2023 Jan 5;12:966088. doi: 10.3389/fonc.2022.966088 (PMC9851621; doi:10.3389/fonc.2022.966088)
Supplement: Supplementary file 1 [file DataSheet_1.docx]

**TABLE S1**. The top 4 most signiﬁcant SF-AS node

| SF | AS | cor | pvalue | Regulation |
| --- | --- | --- | --- | --- |
| EIF3A | MED1\|40644\|AT | 0.759424 | 7.19E-77 | postive |
| PRPF39 | SRSF2\|43663\|RI | 0.744966 | 1.69E-72 | postive |
| LUC7L3 | SRSF2\|43663\|RI | 0.731192 | 1.34E-68 | postive |
| EIF3A | RBM6\|64932\|AT | 0.726822 | 2.06E-67 | postive |


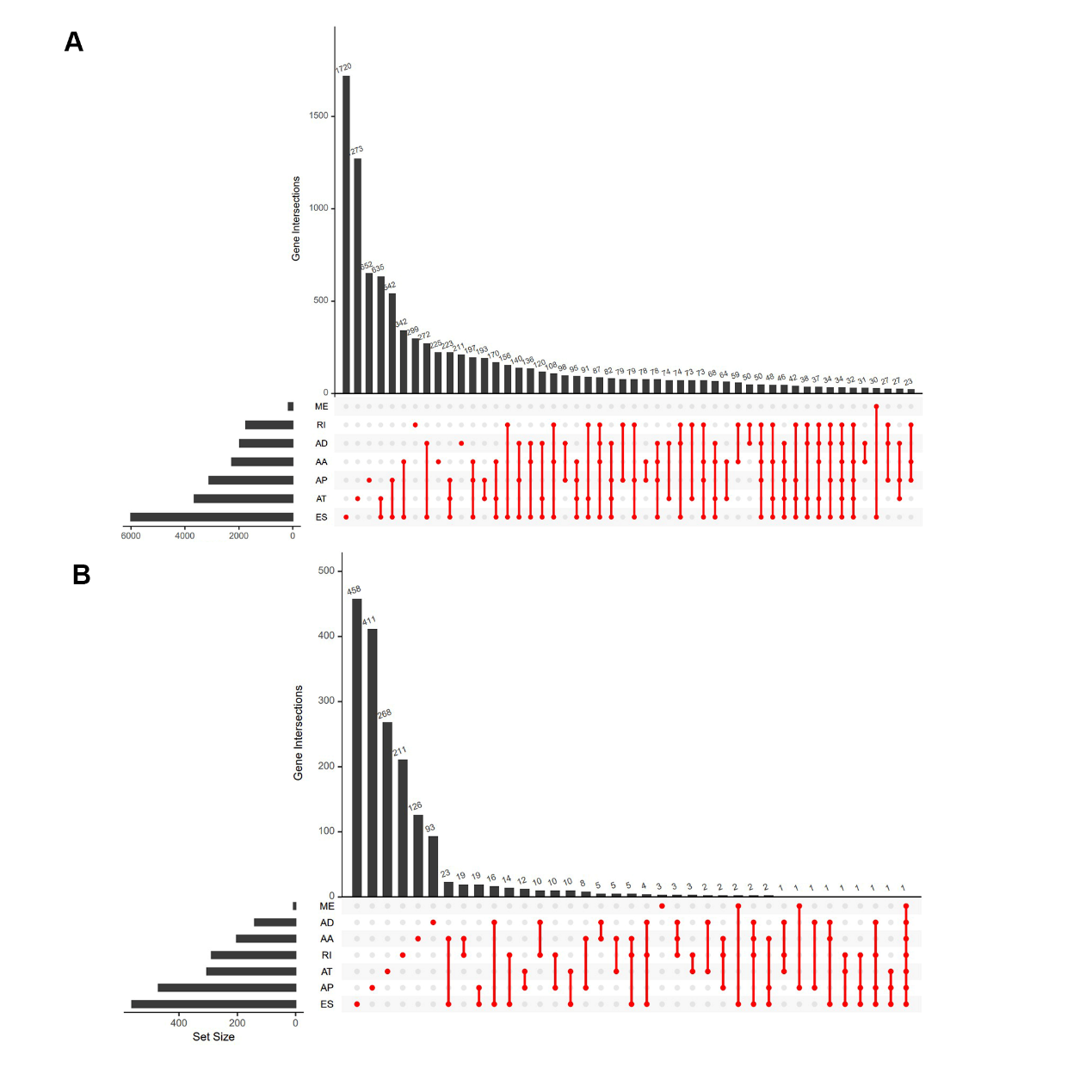


**FigureS1.** (A) Upset plot of alternative splicing events in BLCA. (B) Interaction of alternative splicing events associated with OS in BLCA are displayed in Upset plot.


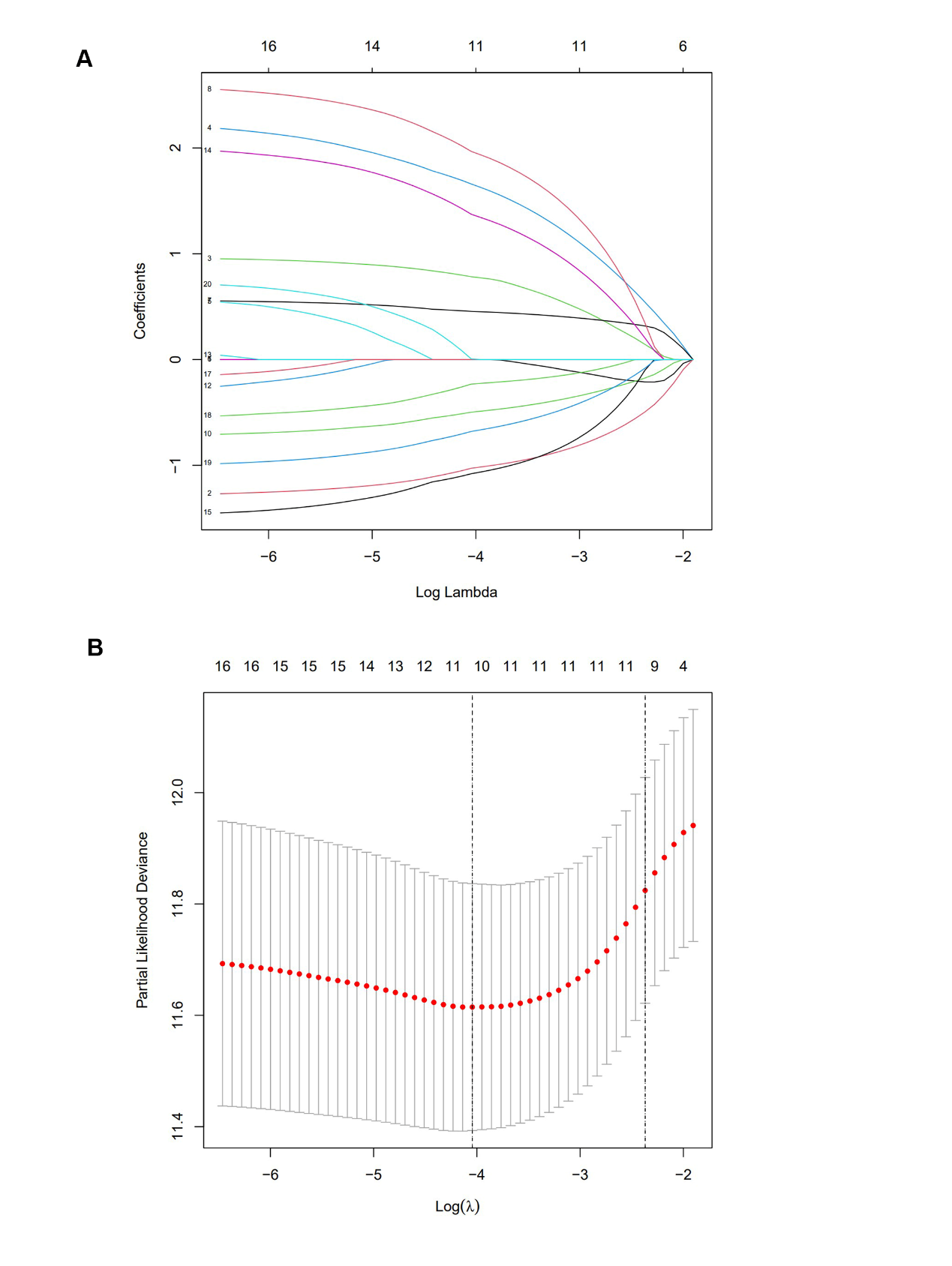


**Figure S2**. The selection of prognostic AS events performed by Lasso regression analysis.


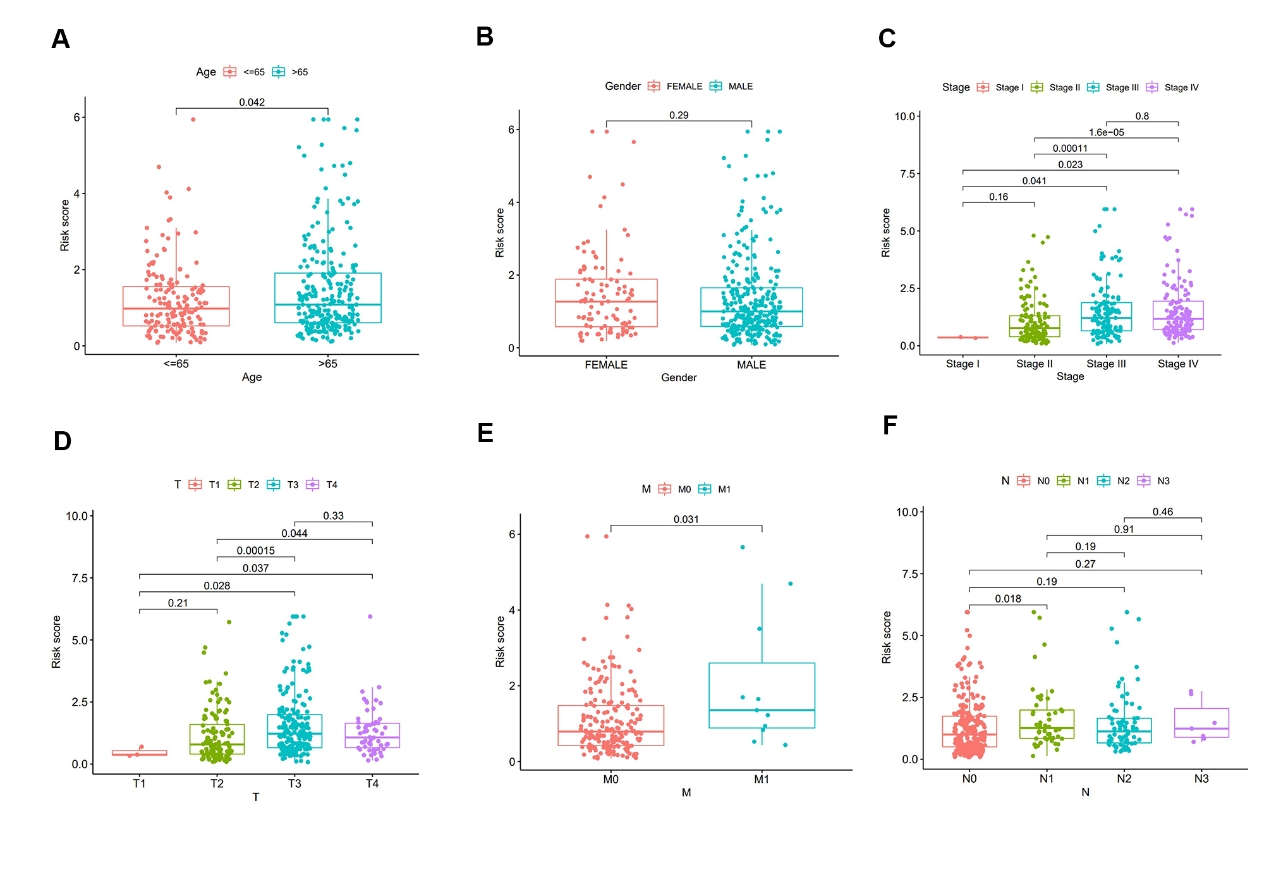


**Figure S3**. Correlation of risk score and clinical parameters. (A) Correlation of risk score and age (B) Correlation of risk score and gender (C) Correlation of risk score and stage (D) Correlation of risk score and T (E) Correlation of risk score and M (F) Correlation of risk score and N.


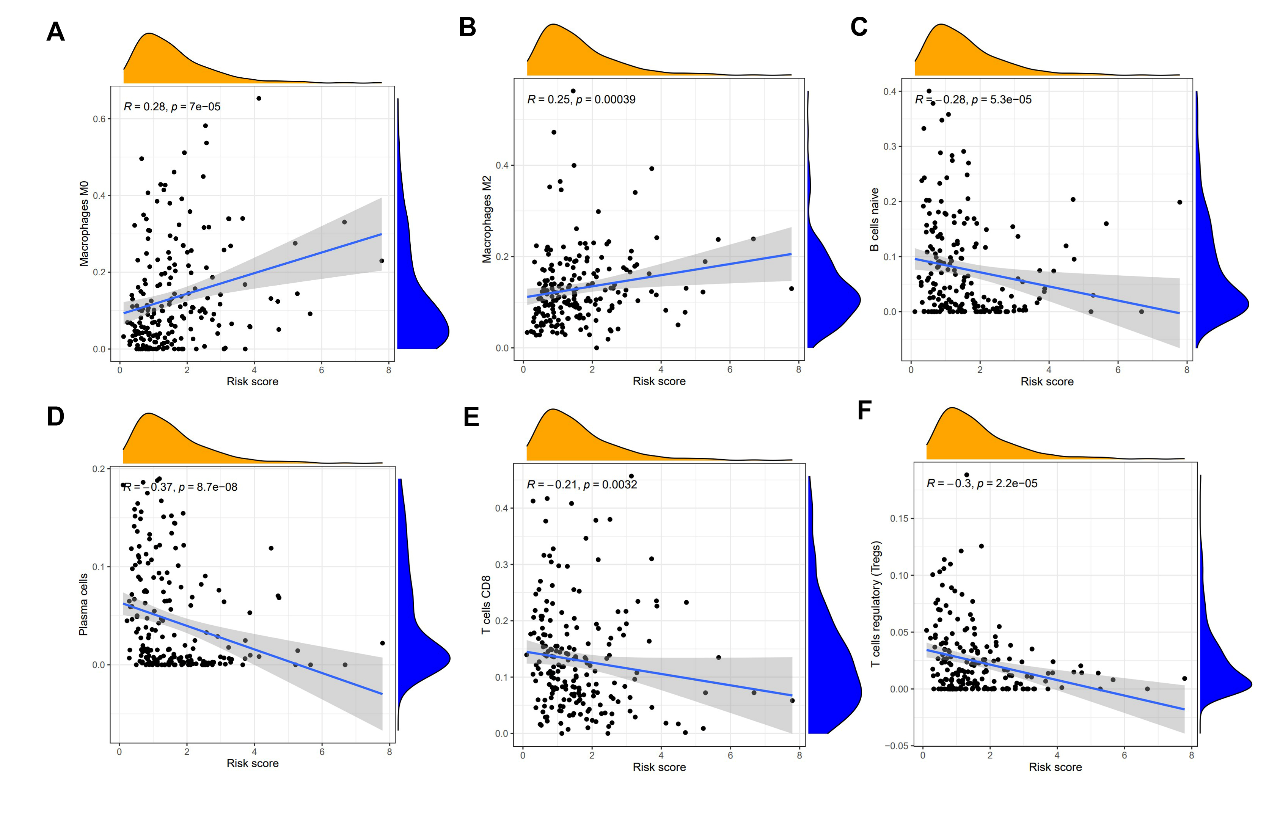


**Figure S4**. Correlation between risk score and immune cells. (A-B) Correlation between risk score and innate immune cells (macrophages M0, M2). (C-F) Correlation between risk score and adaptive immune cells (B cells naïve, plasma cell, CD8 T cell and regulatory T cell).
